# A city-level comparison of fossil-fuel and industry processes-induced CO_2_ emissions over the Beijing-Tianjin-Hebei region from eight emission inventories

**DOI:** 10.1186/s13021-020-00163-2

**Published:** 2020-12-03

**Authors:** Pengfei Han, Ning Zeng, Tomohiro Oda, Wen Zhang, Xiaohui Lin, Di Liu, Qixiang Cai, Xiaolin Ma, Wenjun Meng, Guocheng Wang, Rong Wang, Bo Zheng

**Affiliations:** 1 State Key Laboratory of Numerical Modeling for Atmospheric Sciences and Geophysical Fluid Dynamics, Institute of Atmospheric Physics, Chinese Academy of Sciences, Beijing, China; 2 Department of Atmospheric and Oceanic Science, and Earth System Science Interdisciplinary Center, University of Maryland, College Park, MD USA; 3 Goddard Earth Sciences Research and Technology, Universities Space Research Association, Columbia, MD USA; 4 Global Modeling and Assimilation Office, NASA Goddard Space Flight Center, Greenbelt, MD USA; 5 State Key Laboratory of Atmospheric Boundary Layer Physics and Atmospheric Chemistry, Institute of Atmospheric Physics, Chinese Academy of Sciences, Beijing, China; 6 State Key Laboratory of Pollution Control and Resource Reuse, School of the Environment, Nanjing University, Nanjing, China; 7 Laboratory for Earth Surface Processes, College of Urban and Environmental Sciences, Peking University, Beijing, China; 8 Department of Environmental Science and Engineering, Fudan University, Shanghai, China; 9 Laboratoire Des Sciences du Climat et de L’Environnement, CEA-CNRS-UVSQ, UMR8212, Gif-sur-Yvette, France

**Keywords:** City-level fossil fuel CO_2_, Industry processes, Multiple inventories, Policy making, CO_2_ monitoring

## Abstract

**Background:**

Quantifying CO_2_ emissions from cities is of great importance because cities contribute more than 70% of the global total CO_2_ emissions. As the largest urbanized megalopolis region in northern China, the Beijing-Tianjin-Hebei (Jing-Jin-Ji, JJJ) region (population: 112.7 million) is under considerable pressure to reduce carbon emissions. Despite the several emission inventories covering the JJJ region, a comprehensive evaluation of the CO_2_ emissions at the prefectural city scale in JJJ is still limited, and this information is crucial to implementing mitigation strategies.

**Results:**

Here, we collected and analyzed 8 published emission inventories to assess the emissions and uncertainty at the JJJ city level. The results showed that a large discrepancy existed in the JJJ emissions among downscaled country-level emission inventories, with total emissions ranging from 657 to 1132 Mt CO_2_ (or 849 ± 214 for mean ± standard deviation (SD)) in 2012, while emission estimates based on provincial-level data estimated emissions to be 1038 and 1056 Mt. Compared to the mean emissions of city-data-based inventories (989 Mt), provincial-data-based inventories were 6% higher, and national-data-based inventories were 14% lower. Emissions from national-data-based inventories were 53–75% lower in the high-emitting industrial cities of Tangshan and Handan, while they were 47–160% higher in Beijing and Tianjin than those from city-data-based inventories. Spatially, the emissions pattern was consistent with the distribution of urban areas, and urban emissions in Beijing contributed 50–70% of the total emissions. Higher emissions from Beijing and Tianjin resulted in lower estimates of prefectural cities in Hebei for some national inventories.

**Conclusions:**

National-level data-based emission inventories produce large differences in JJJ prefectural city-level emission estimates. The city-level statistics data-based inventories produced more consistent estimates. The consistent spatial distribution patterns recognized by these inventories (such as high emissions in southern Beijing, central Tianjin and Tangshan) potentially indicate areas with robust emission estimates. This result could be useful in the efficient deployment of monitoring instruments, and if proven by such measurements, it will increase our confidence in inventories and provide support for policy makers trying to reduce emissions in key regions.

## Background

Cities play a significant role in global greenhouse gas emissions, especially in urban areas, which are responsible for 67–76% of the global CO_2_ emissions and energy consumption [1]. Cities have become the critical and basic units for implementing emissions mitigation policies [2–5]. However, city-level mitigation actions remain daunting challenges [1, 6, 7]. City carbon emissions are influenced by the physical environment, economic development, urbanized density, industry structure, and energy use patterns specific to each city [1, 8]. Cities with a heavy industry, high traffic load, and high population density more easily have high emissions [3]. Discrepancies in the emissions and emission-socioeconomic characteristics among different cities require the development of corresponding policies [4]. Moreover, how to deploy observational instruments to form an efficient network is rather challenging [9–11], especially when there is no robust understanding of emissions. With emissions patterns identified by multiple inventories, such deployments would have more scientific guidelines. Furthermore, city strategies that reduce carbon emissions are expected to achieve emissions mitigation and meet a city’s economic growth goals [12]. Therefore, an accurate understanding of city-level CO_2_ emissions is of great importance in developing and implementing mitigation strategies.

With rapid economic development and urbanization in China, cities account for 85% of China’s CO_2_ emissions [13]. Emission error could be much larger at subnational levels [14]. However, most of the existing studies focus on national [15–19], provincial [4, 16, 20, 21], or sectoral CO_2_ emissions inventories in China [22–25]. For example, using nine inventories, Han et al. [19] estimated that the national total fossil fuel and industrial process-related CO_2_ emissions were 9.8 (9.2–10.4) Gt CO_2_ in 2016, and the emissions estimated from provincial-data-based inventories were more consistent than those from spatial disaggregation of national energy statistics. A few efforts have been made to estimate city-level emissions, but these efforts have mainly focused on megacities or provincial capital cities due to the limited energy data [26–29]. To date, the studies by Shan et al. [12] and Cai et al. [30] included 182 and 305 Chinese cities, respectively, and still had gaps in city coverage. Zheng et al. [31] estimated all the cities’ CO_2_ emissions intensities in mainland China, yet the latest year included in the study was 2013, and the study lacked temporal dynamics. Thus, a more comprehensive assessment of city-level CO_2_ emissions in China is critical for understanding the role of cities in carbon emissions.

The Beijing-Tianjin-Hebei (Jing-Jin-Ji, JJJ) region is the largest urbanized megalopolis region in northern China, covering an area of 218,000 km^2^ and home to 112.70 million people [32–34]. Cities in the JJJ region include the municipalities Beijing and Tianjin and eleven prefecture cities in Hebei Province. In 2018, the total energy consumption in the JJJ region accounted for more than 10% of China’s total energy consumption [34]. Moreover, coal is the primary energy source in this region [33, 34]. The JJJ region is under considerable pressure to reduce CO_2_ as well as air pollutant emissions [31, 35]. Beijing and Tianjin have committed to peak their CO_2_ emissions by 2020 and approximately 2025, respectively, in the 13th Five-Year Plan [36]. Hebei Province is experiencing rapid industrial and urban development [37], contributing greatly to the national CO_2_ emissions [4]. However, there is a wide range of CO_2_ emission estimates in the JJJ region, especially in the areas with high emissions. The emissions from Beijing and Tianjin estimated by Cai et al. [30] and Wang [38] were 41–57% higher than those estimated by Shan et al. [16]. In addition, based on the results of Mi et al. [39] and Cai et al. [30], Tangshan’s carbon emissions differed by 50%. To our knowledge, there is not a comprehensive assessment on prefectural city level CO_2_ emissions in the JJJ region. Cai et al. [33] reported the provincial emissions, but not covered the prefectural cities in JJJ. Therefore, an accurate estimation of the CO_2_ emissions in the JJJ region is of great significance in terms of providing accurate information for developing mitigation policies.

The assessment of existing CO_2_ emissions inventories is urgently needed, yet direct observations of CO_2_ emissions at the city scale is limited, especially in developing countries [10, 11]. Here, we conducted a comprehensive analysis of 8 state-of-the-art inventories and presented the temporal dynamics, spatial distributions, and urban and non-urban fractions of 13 cities. We recognized the similarities and differences in emissions and thus improved the understanding of current inventories; this research provides useful information for policy making related to reducing city emissions and monitoring CO_2_.

## Data and methods

### Data

We used annual CO_2_ emissions data from eight emission inventories, including the China High Resolution Emission Database (CHRED); China Emission Accounts and Datasets (CEADs); Multi-resolution Emission Inventory for China (MEIC), version 1.3; the Nanjing University CO_2_ emission inventory (NJU); the Peking University CO_2_ emission inventory (PKU), version 2 (PKU-CO_2_-V2); the Open-source Data Inventory for Anthropogenic CO_2_, version 2018 (ODIAC2018); the Emissions Database for Global Atmospheric Research, version 5.0 (EDGARv5.0); and the Fossil Fuel Data Assimilation System, version 2.2 (FFDAS v2.2). Below, the eight inventories were categorized into two based on the emission calculation methodologies (Table 1).

**Table 1:** Summary of the emission inventories ( modified from Han et al. [19])

Data	ODIAC	EDGAR	PKU-CO2	FFDAS	CHRED	MEIC	NJU	CEADs
Domain	Global	Global	Global	Global	China	China	China	China
Temporal coverage	2000–2016	1970–2012	1960–2014	1997–2015	2007, 2012	2000–2016	2000–2015	1997–2015
Temporal resolution	Monthly	Annual	Monthly	Hourly/Annual	Biennially or triennially	Monthly	Annual	Annual
Spatial resolution	1 km	0.1 degree	0.1 degree	0.1 degree/ 1 km	10 km	0.25 degree	0.25 degree	N/A
Emission estimates	Global/National	Global/ National	Global/National	Global/National	National/Provincial	National/Provincial	National/Provincial	Prefectural/National/ Provincial
Emission factor for raw coal (tC per t of coal)	0.746	0.713	0.518	–	0.518	0.491	0.518	0.499
National uncertainty	17.5% (95% CI)	± 15%	± 19% (95% CI)	5- 15%	± 8%	± 15%	7–10% (90% CI)	−15%–25% (95% CI)
Point source	CARMA2.0	CARMA3.0	CARMA2.0	CARMA2.0	FCPSC	CPED	CEC;ACC;CCTEN	N/A
Line source	N/A	The OpenStreetMap and OpenRailwayMap, Int. aviation and bunker	N/A	Transport networks	The national road, railway, navigation network, and traffic flows	Transport networks	N/A	N/A
Area source	Nighttime light	Population density, nighttime light	Vegetation and population density, nighttime light	Nighttime light	Population density, land use, human activity	Population density, land use	Population density, GDP	N/A
Version name	ODIAC2018	EDGARv50_	PKU-CO2-v2	FFDAS v2.2	CHRED	MEIC v.1.3	NJU-CO2v2017	CEADs
Year published/updated	2018	2019	2016	2014	2017	2018	2017	2017
Data sources	http://db.cger.nies.go.jp/dataset/ODIAC/	https://edgar.jrc.ec.europa.eu/overview.php?v=50_GHG	http://inventory.pku.edu.cn/download/download.html	http://ffdas.rc.nau.edu/Data.html	Data developer	Data developer	Data developer	http://www.ceads.net/
References	Oda [40]	Janssens-Maenhout [41]	Wang et al. [51]	Asefi‐Najafabady et al. [42]	Cai et al. [43]; Wang et al. [44]	Zheng [45]; Liu et al. [46]	Liu [47]	Shan et al. [48]; Guan et al. [17]

### Emission inventories based on city-level data

We used two inventories that are based on city-level data. CHRED was constructed by enterprise-level point-source data and China’s city and provincial statistics, and then carbon emissions were allocated to 10 km resolution using proxy data in 2007 and 2012 [15, 30]. The CEADs inventory provides total CO_2_ emission estimates at the provincial and city levels from 2000 to 2016 based on apparent energy consumption data and local optimized emission factors [4, 17, 49].

### Emission inventories based on provincial or national level data

We used two inventories that are based on province-level data. China’s carbon emissions from MEIC are developed by a technology-based methodology based on provincial energy consumption, combustion/industrial/control technologies and emission factor databases covering 2000 to 2016 at 0.25, 0.5, and 1 degree spatial resolutions by Tsinghua University [18, 31, 50]. NJU calculated China’s CO_2_ emissions using provincial energy statistics and spatially distributed emissions based on the location of large point sources (power plants and cement plants) and various proxy data, i.e., using GDP for industry-related emissions and population for transportation and other emissions, at 0.25 degree resolution from 2000 to 2016 [5, 18].

We used 4 inventories that are based on national-level emission estimates. These subnational emission distributions are largely achieved by emission downscaling. PKU distributes national or subnational fuel data with various proxies (e.g., power plants as point sources, night-time light to distribute national gas flaring and population for others) based on the subnational disaggregation method at 0.1 degree resolution from 1960 to 2014 [51]. The year 2018 version of the ODIAC emissions data product (ODIAC2018) distributes national emissions into 1 km and 1 degree grids from 2000 to 2017 based on spatial proxies, such as geographical locations of power plant emissions, satellite observations of nightlights to distribute nonpoint emissions, and aircraft and ship fleet tracks [52, 53]. EDGAR provides carbon emissions on the 0.1 degree grid from 1970 to 2018 based on national emissions by a variety of spatial proxy data, including power plants from CARMA3.0 for point sources, road network and different weighting factors for line sources and population for residential and commercial emissions [54, 55]. FFDAS quantifies carbon emissions using a data assimilation technique incorporating remote-sensing and national fuel accounts and power plants at a 0.1 degree resolution from 1997 to 2015 [42, 56]. All data sets used here have yearly data. For more details, please refer to Han et al. [19].

### Methods

These inventories were first extracted by a JJJ mask (in shapefile format) from the National Geomatics Center of China using ArcGIS 10.02 software (ESRI, 2012). The emissions from urban and non-urban areas were separated by using an urban mask from the European Space Agency (ESA) Climate Change Initiative (CCI) land cover maps with a 300 m resolution (https://www.esa-landcover-cci.org), and the urban area here mainly refers to impermeable surfaces, with high coherence (stability) and bright reflections, maintained in time and under varying angles detected by satellite. This means there is a density of human structures such as houses, commercial buildings, roads, bridges, and railways, while a city refers to prefectural level zones with a legal definition that defines a physical geographic boundary. ArcGIS was used to obtain prefectural cities’ total emissions in urban and non-urban areas using city mask data from the National Geomatics Center of China. The data pre-process procedures are as follows: (1) Convert the grid data to polygon data, which will keep the original value of the grid; (2) Split the polygon data using the prefectural city boundaries, and this step produces the real areas of a polygon within the city boundary; (3) Calculate the actual areas of each polygon using “Calculate geometry” and multiply the areas with emissions intensity (e.g. kg CO_2_/km^2^); (4) Finally, sum all the values within the boundary of a city. Emission intensity was calculated as the CO_2_ emissions divided by the GDP, data which were derived from the National Bureau of Statistics of the People's Republic of China (NBS) and websites of prefectural cities’ statistics bureaus in Hebei. Linear regressions were conducted using the Python scipy package between inventories with CEADs or CHRED.

## Results

### City-level CO_2_ emissions

**Fig. 1:**
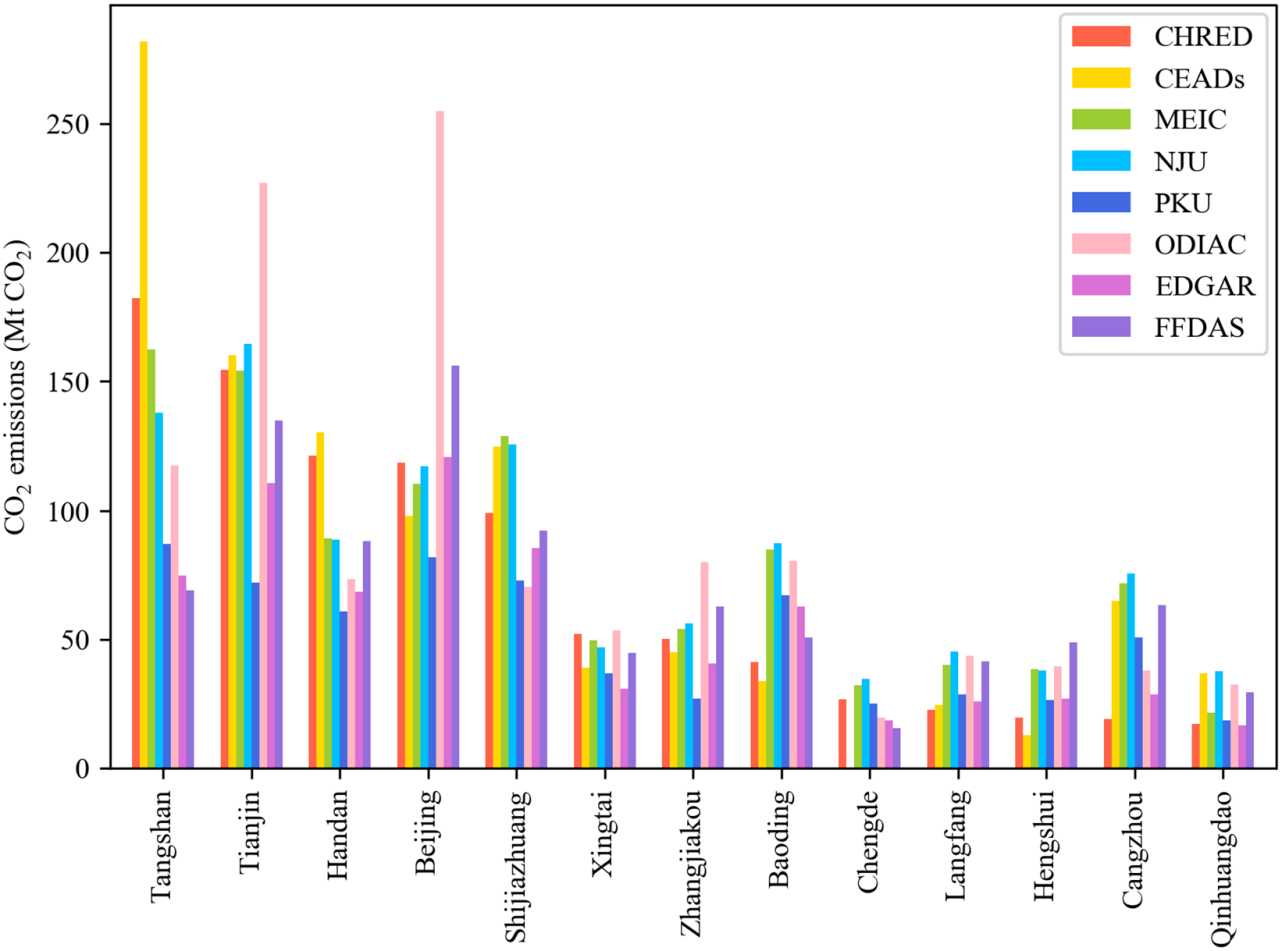
City-level CO_2_ emissions from CHRED, CEADs, MEIC, NJU, PKU, ODIAC, EDGAR, and FFDAS in the Beijing-Tianjin-Hebei (JJJ) region in 2012

In 2012, the emissions of 13 cities varied widely from 13 to 282 Mt CO_2_ (or 72 ± 45, mean ± SD) (Fig. 1). Tangshan, Tianjin, Handan and Beijing are high-emission areas. The total emissions estimated from provincial-data-based inventories (i.e., MEIC and NJU) were 6% higher than those from city-data-based inventories (i.e., CHRED and CEADs) but were 14% lower from downscaled national-level emissions (i.e., PKU, ODIAC, EDGAR, and FFDAS) in 2012. There was a great discrepancy in national-data-based inventories with a range from 657 to 1132 Mt CO_2_ (or 849 ± 214, mean ± SD). The CO_2_ emissions from EDGAR and PKU were 28% and 34% lower than the average from city-data-based inventories. The 13 cities have substantial differences in natural resources and socioeconomic conditions. A city with high productivity, rapid economic growth, and a large population tends to have high carbon emissions. Emissions from metropolises (e.g., Beijing and Tianjin) with advanced economics and high urbanization rates greatly contributed to the JJJ total emissions (23–43%, Fig. 1). In Hebei Province, high emissions were also located in the provincial capital and in industrial cities, such as Shijiazhuang, Tangshan, Handan, and Baoding, which accounted for 57%–68% of the total emissions in Hebei. The 7 remaining cities accounted for 32–43% of the total emissions in Hebei.

Since CHRED and CEADs use city-level statistical data and these two datasets are more consistent with each other than with the other datasets, they are used as references for national and provincial data-based inventories. The emissions of Beijing from EDGAR, MEIC, and NJU are comparable to those from CHRED, with differences ranging from 1 to 8 (1–7%) Mt CO_2_ in 2012. However, compared to the values estimated by CHRED, the emissions were 115% and 32% higher when estimated by ODIAC and FFDAS, respectively, and 31% lower when estimated by PKU. These trends were also found in Tianjin, and the emissions from ODIAC were equal to 227 Mt CO_2_, a value that was 47% higher than that from CHRED but 53% lower than that from PKU. Emissions in prefectural cities with coal mines and heavy-intensity industries also showed large differences, such as Tangshan and Handan. Tangshan’s emissions varied largely across these datasets. CHRED and CEADs both produced larger estimates (182 and 282 Mt), while estimates from PKU, EDGAR, and FFDAS were only 69–87 Mt, or 52–62% lower, than that by CHRED. Handan’s emissions were up to 121 and 130 Mt based on CHRED and CEADs, respectively, but ranged from 61 to 89 Mt (26–53% lower than CHRED and CEADs) for the other inventories. It should also be noted that CHRED and CEADs produced large differences in some cities, such as Tangshan, Shijiazhuang, and Cangzhou, and these differences highlighted future directions for city-level inventories in JJJ. According to the plan of “Collaborative Development of Beijing, Tianjin and Hebei Province”, Zhangjiakou and Chengde belong to ecological conservation areas; thus, heavy industry is not recommended, and more efforts should be concentrated in high-emission cities such as Tangshan, Shijiazhuang and Handan.

### Spatial pattern of CO_2_ emissions

**Fig. 2:**
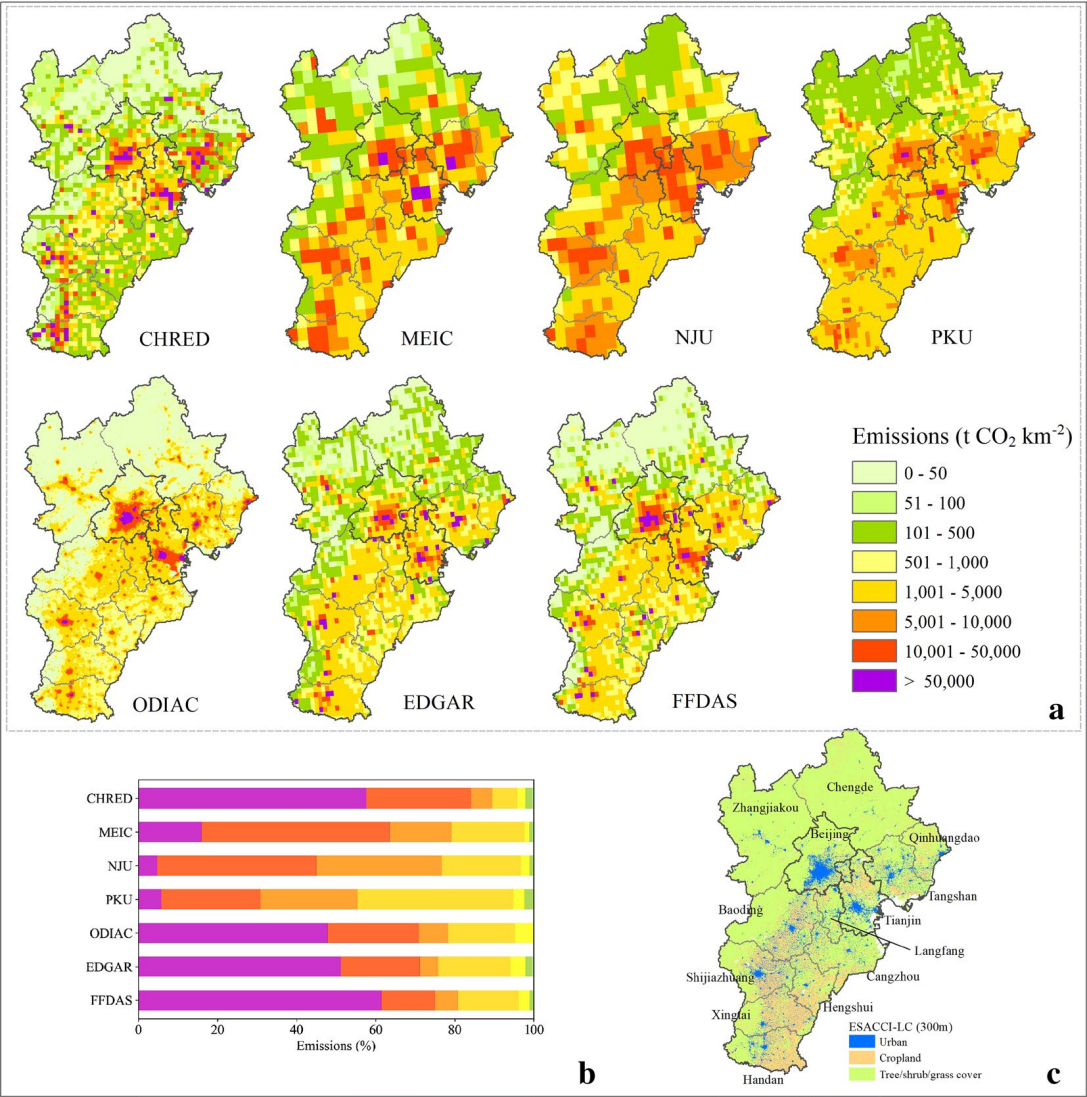
Spatial distribution of CO_2_ emissions (**a**) from CHRED, MEIC, NJU, PKU, ODIAC, EDGAR, and FFDAS in the JJJ region in 2012. Horizontal bars represent emissions fractions from each category of total emissions (**b**). Urban, cropland and tree and shrub cover from ESA_CCI_LC

The uneven spatial distribution of CO_2_ emissions reflects the highly diverse conditions in population, economic development, and natural environment in the JJJ region. The spatial distributions showed reasonably good agreement in patterns such as the high emissions areas of Beijing-Tianjin-Tangshan, although they varied in detail (Fig. 2). Low emissions (e.g., < 100 ton CO_2_ km^−2^) from different datasets were mainly located in the northwestern part of the region. This result is because cities in the north, such as Zhangjiakou and Chengde, have a low population density and have lower economic development [39]. High-emission areas (> 10,000 t CO_2_ km^−2^) are clustered in urban centers in the south and east. Hotspots of CO_2_ emissions (> 50,000 t CO_2_ km^−2^) recognized by most inventories are located in the urban areas of Beijing, Tianjin, and Tangshan.

Specifically, the spatial distributions of CO_2_ emissions from EDGAR and FFDAS are consistent with the pattern from CHRED. However, the total amount of emissions in the region from EDGAR is 23% lower than that from CHRED. This difference is caused by the discrepancies estimated in Handan, Tianjin, and Tangshan from EDGAR, which is altogether approximately 204 Mt CO_2_ lower than that from CHRED. Although the difference in total emissions between FFDAS and CHRED is less than 3%, the emissions in Tangshan and Beijing from FFDAS are 62% lower and 32% higher than those from CHRED. ODIAC distributes the non-power plant portion of national emissions based on satellite nighttime light [52, 53]. The pattern of carbon emissions in ODIAC is highly consistent with the spatial distribution of urban areas. Low emissions ranging from 50 to 500 t CO_2_ km^−2^ are not found in ODIAC. This result is probably due to the low emissions mostly located in the non-urban areas, which are mainly covered by vegetation and do not emit strong night light (Fig. 2). ODIAC and FFDAS have similar patterns, shown in Fig. 2, which can be because they both used night-time light to distribute nonpoint emissions. Compared with CHRED, the carbon emissions for the remaining inventories all showed higher emissions in the southeastern areas, which are mainly covered by croplands, with the results of MEIC, NJU, PKU and ODIAC being more notable and the results of EDGAR and FFDAS being relatively weaker. For the spatial allocation of emissions, CHRED, EDGAR and FFDAS give more emissions to high emitting grids, with > 50,000 t CO_2_/km^2^ grids contributing more than 50% of the total emissions (Fig. 2c), while for NJU and PKU, the same level of high emitting grids contributed less than 10%.

**Fig. 3:**
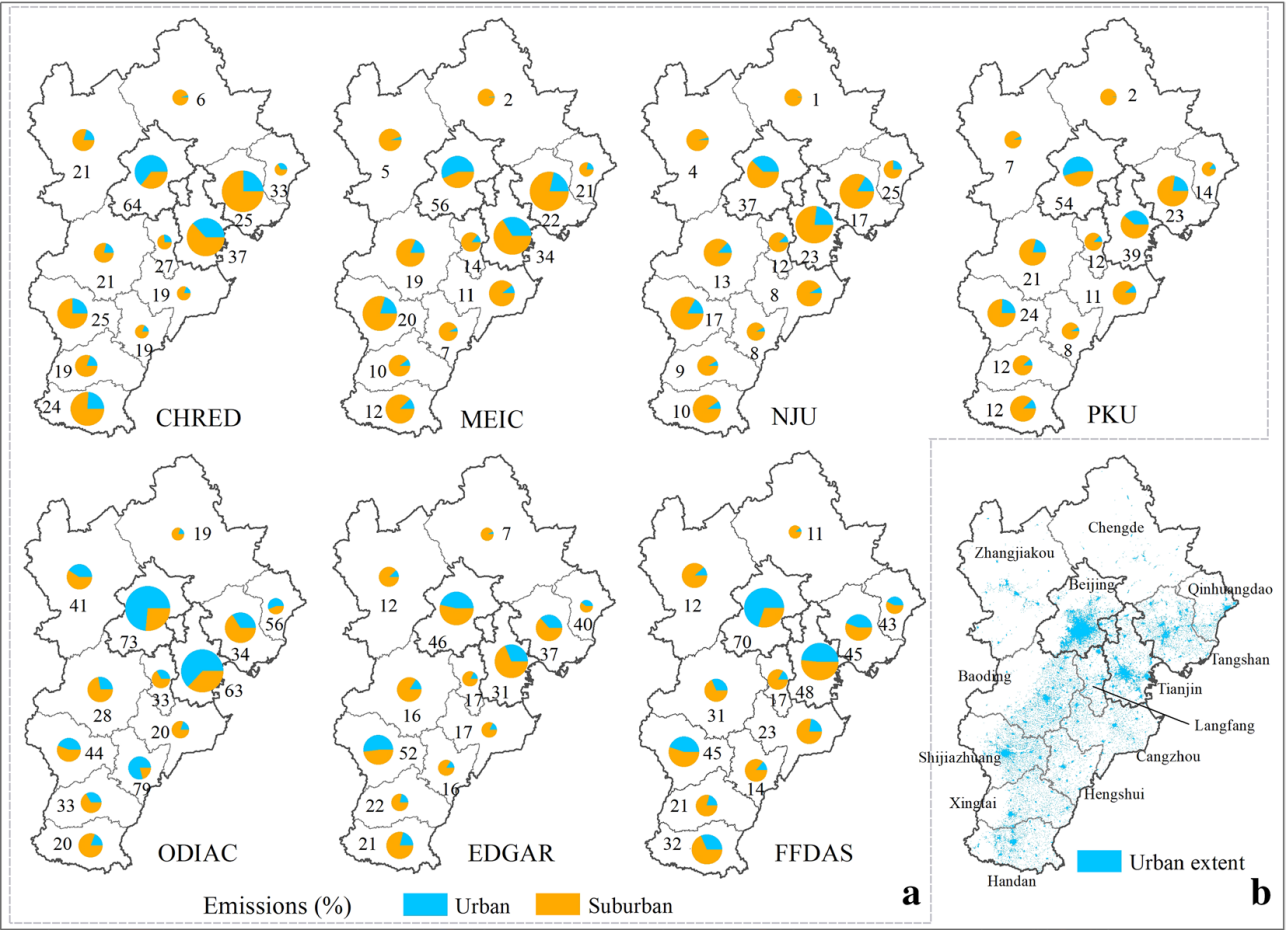
Spatial distribution of urban and non-urban CO_2_ emissions from CHRED, MEIC, NJU, PKU, ODIAC, EDGAR, and FFDAS in the JJJ region in 2012 for subplot (**a**). The area of the pie chart represents the amount of CO_2_ emissions per city. The number near each pie chart represents the urban emission fraction. And the urban extent from ESA_CCI_LC for subplot (**b**)

It should be noted that the urban area contributed largely to the total emissions because of its large energy consumption and population [57]. Here, according to the urban extent from the ESA CCI land cover maps with a 300 m resolution (https://www.esa-landcover-cci.org) (Fig. 2), we extracted urban emissions from the seven gridded inventories, namely, CHRED, NJU, MEIC, PKU, ODIAC, EDGAR and FFDAS. As illustrated in Fig. 3, the proportion of urban emissions shows obvious differences among datasets, ranging from 17% (NJU) to 50% (ODIAC). As the largest contributors, the proportions of urban emissions in Beijing and Tianjin varied between 37% (NJU) –73% (ODIAC) and 23% (NJU) –63% (ODIAC), respectively. ODIAC tended to overestimate emissions in urban areas, resulting in the strongest urban-non-urban emissions gradients, especially in Tianjin (63% from urban) and Hengshui (79% from urban). This result is due mainly to the use of the nightlight proxy [14, 58]. The urban emissions from FFDAS also show a similar pattern with those from ODIAC, except for Zhangjiakou and Hengshui. In addition, in high-emitting cities in Hebei, such as Tangshan, all inventories identified urban emissions accounting for a smaller fraction (20%–40%) than those in Beijing and Tianjin.

### Temporal variation in city-level CO_2_ emissions and emissions intensity

**Fig. 4:**
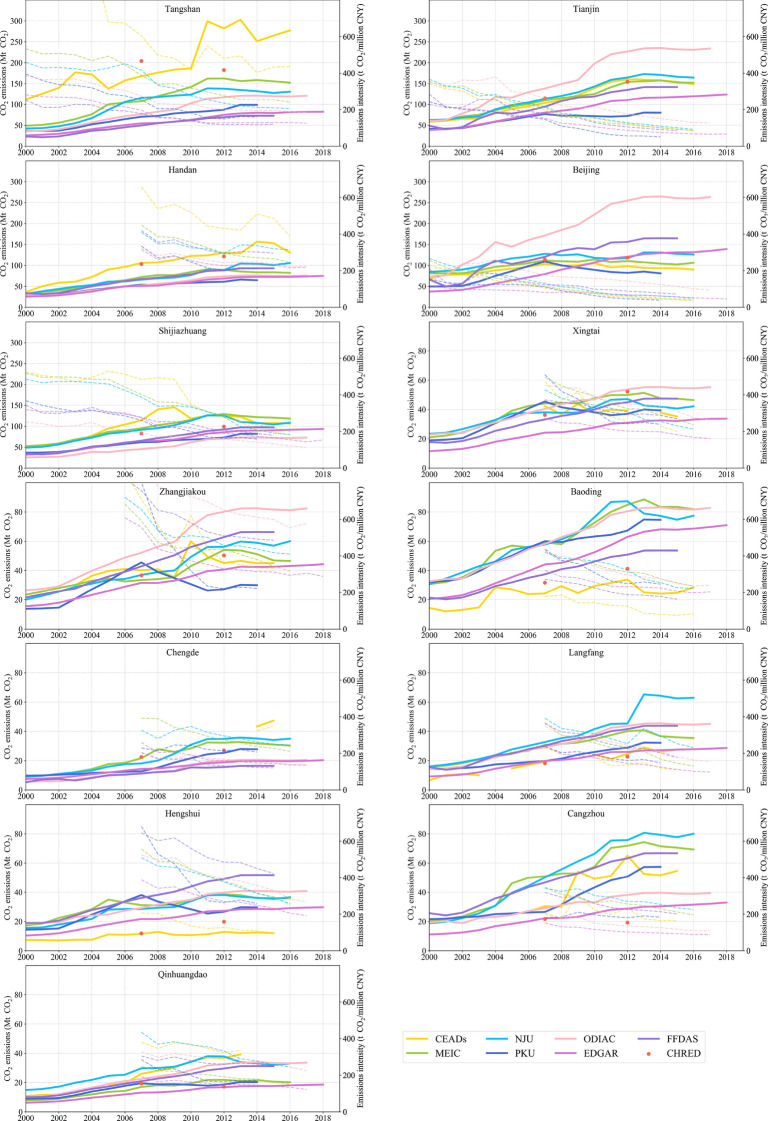
Temporal variation in the annual CO_2_ emission amounts and emissions intensity from CHRED, CEADs, MEIC, NJU, PKU, ODIAC, EDGAR, and FFDAS in the JJJ region. Solid lines denote CO_2_ emissions, and dashed lines denote emissions intensity. Note that the scales are different across cities to show their variations

The interannual variations in carbon emissions from existing inventories all showed an increasing trend during 2000–2012, ranging from 355 ± 58 Mt to 915 ± 178 Mt CO_2_, and then tended to level off or showed a slight downward trend afterwards (Fig. 4). Total emissions of the JJJ region from ODIAC, CEADs and EDGAR increased faster than others, with average growth rates of 9.7%, 9.2% and 9.1%, respectively, during 2000–2012. However, the regional total emissions from FFDAS, PKU, MEIC, and NJU showed a relatively small trend, with an annual growth rate of 6.0–8.4%. PKU and EDGAR tended to underestimate emissions compared to CHRED and CEADs, especially in high-emitting cities. The interannual variation at the city scale from MEIC was consistent with that from NJU, both of which were based on provincial statistical data.

Specifically, for Beijing, the CO_2_ emissions from MEIC, NJU, CEADs, PKU, and CHRED tended to be stable since 2007, which was consistent with the studies by Li et al. [59] and Shan et al. [60]. This result may be because Beijing decreased coal use by 43% and increased natural gas consumption by 144% from 2007 to 2014 [4, 16]. However, the annual emissions in Beijing from ODIAC fluctuated greatly, ranging from 73 Mt CO_2_ in 2000 to 263 Mt CO_2_ in 2017. PKU is a downscaled inventory; however, it uses provincial consumption fractions to rescale the IEA (International Energy Agency) total fossil fuel consumption when distributing emissions to grids, and thus, it can capture the Beijing decreasing trend after 2007, while other national downscaled inventories cannot. The emissions in Tianjin and Tangshan experienced rapid growth and then outpaced the emissions of other cities in recent years. The time series of emissions in Tianjin from ODIAC, MEIC, NJU, CEADs, and CHRED all showed an apparent growing trend during 2009–2014, which mainly resulted from the increment of coal use (22%) and crude oil (90%) [61]. The decline between 2005 and 2010 for CEADs was because of the change in statistical methods and dimensions, and blast furnace gas was lacking from the energy balance sheet during this period. For other periods, high emissions and large fluctuations in Tangshan’s emissions from CEADs were partially caused by its heavy industrial system [12, 39], whose iron and steel production accounted for 60–70% of Hebei’s total production.

The emissions intensity (CO_2_ emissions per unit of GDP) in the JJJ region showed a decreasing trend since 2000 (Fig. 4). Interannual changes in emissions intensity among datasets were consistent with the total emissions. Moreover, the decoupled relationship between an increase in GDP and a decrease in emissions intensity indicated that a reduction in carbon emissions intensity could be achieved while also maintaining economic growth. For example, the GDP in Beijing and Tianjin significantly increased by average annual rates of 14% and 16%, respectively, during 2000–2016, but emissions intensity declined by 8–11% annually from PKU, MEIC, NJU, and CEADs and by 5–7% from ODIAC, EDGAR, and FFDAS.

### Comparison of city-level CO_2_ emissions from different inventories to CHRED and CEADs

**Fig. 5:**
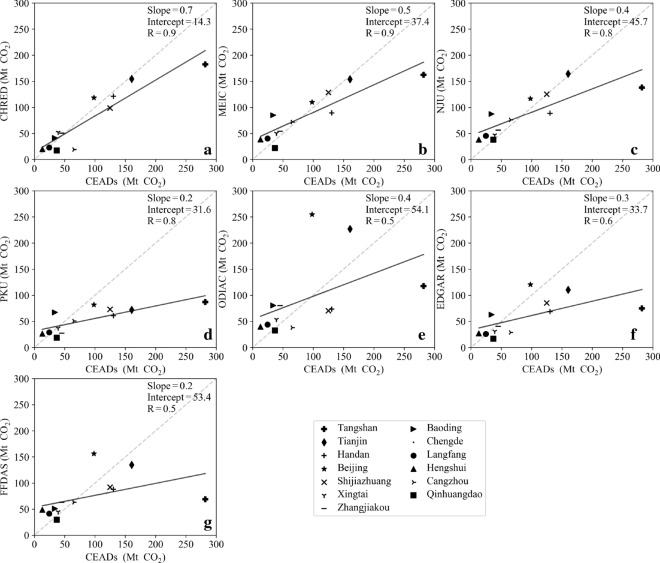
Correlation of city-level emissions from CEADs and from CHRED (**a**), MEIC (**b**), NJU (**c**), PKU (**d**), ODIAC (**e**), EDGAR (**f**), and FFDAS (**g**) in the JJJ region

**Fig. 6:**
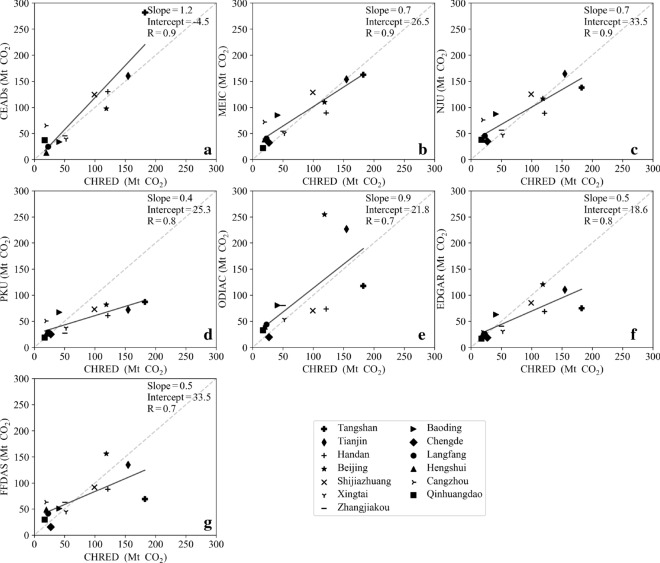
Correlation of city-level emissions from CHRED and from CEADs (**a**), MEIC (**b**), NJU (**c**), PKU (**d**), ODIAC (**e**), EDGAR (**f**), and FFDAS (**g**) in the JJJ region

CHRED and CEADs were used as references to evaluate other datasets because they are both based on city-level energy statistics and consistent with each other. Furthermore, CHRED included unique comprehensive point sources (over 1.5 million enterprises) [33], and CEADs used measured local emission factors from 602 coal samples and 4243 coal mines [49]. As expected, the city-level CO_2_ emissions showed the best correlation between CEADs and CHRED, with a correlation coefficient (R) as high as 0.9 (Figs. 5a and 6a) and with a slope close to 1 and a smaller intercept than the others. The emissions from MEIC and NJU were both highly correlated with those from CEADs and CHRED, with R values ranging from 0.8 to 0.9 and slopes ranging from 0.5 to 0.7. These results were probably due to MEIC and NJU using provincial energy statistics as input data. CHRED and CEADs also used provincial inventories to calculate some city-level CO_2_ emissions because of the lack of consistent and accurate energy statistics in certain cities [12, 30].

PKU was strongly correlated with CEADs and CHRED, with R values both equal to 0.8. The slopes were only 0.2 and 0.4 for the correlation relationships of PKU and CEADs and of PKU and CHRED, respectively (Figs. 5 and 6). The lower emissions estimated from PKU were mainly due to the rescaled energy data for China’s total by IEA statistics. Emissions from EDGAR had a relatively weak relationship with those of CEADs and CHRED, with R values of 0.6 and 0.8, respectively. ODIAC and FFDAS showed the lowest relationships with CEADs and CHRED, with R values of 0.5 and 0.7, respectively. These results were probably due to ODIAC, EDGAR and FFDAS all disaggregating emissions based on national energy statistics.

## Discussions

### Differences in prefectural-city-, provincial- and national-statistical data-based inventories

**Fig. 7:**
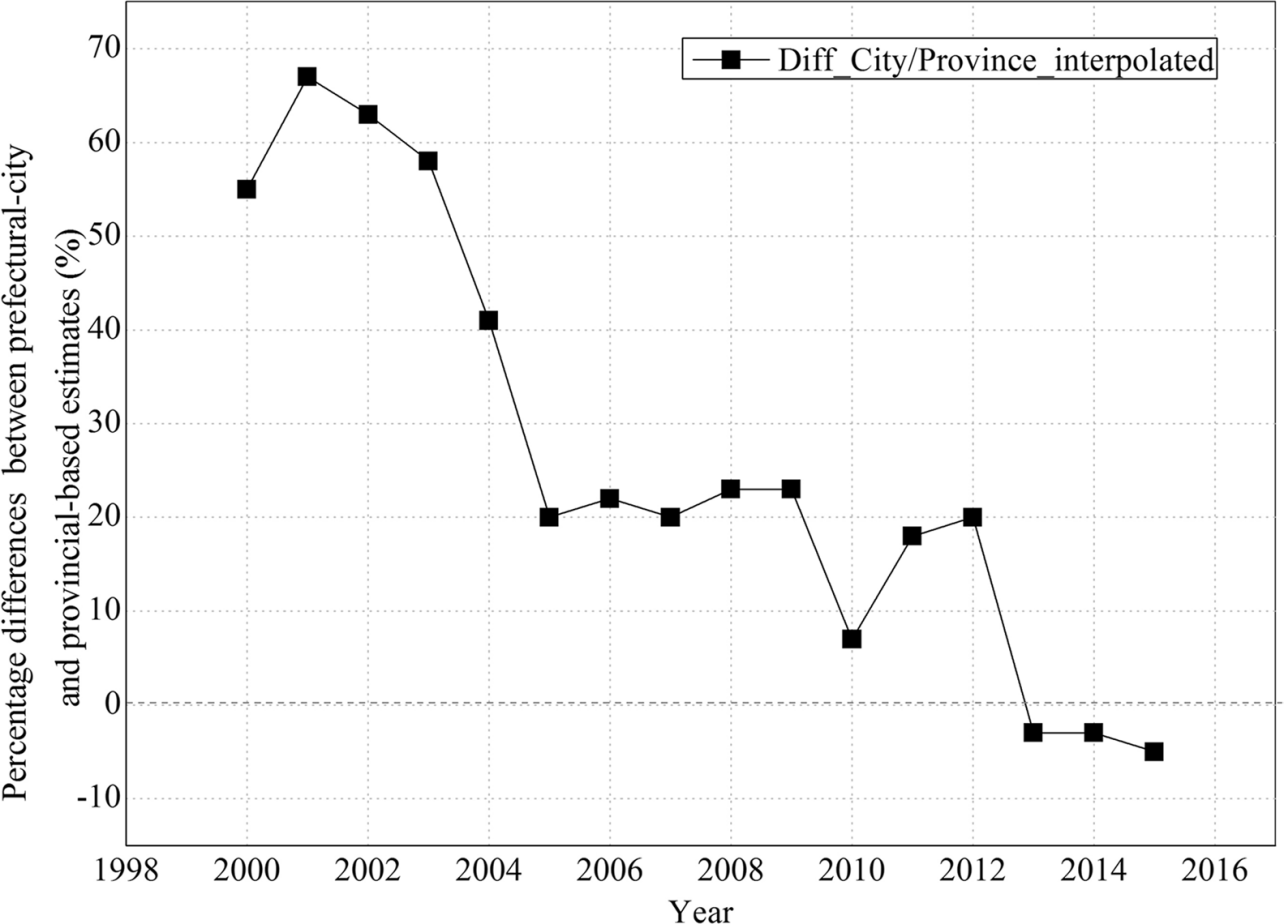
Percentage differences between prefectural-city-based and provincial-based estimates for CEADs. For the years without data, values were interpolated using data from nearby years

The differences between provincial- and national-statistical data-based emissions have been well discussed in previous studies [19, 62, 63]. However, the differences between provincial and prefectural city statistical data-based emissions are poorly understood. Thus, it is difficult to conclude which is closer to the truth. Of these inventories, CEADs provided both prefectural-city- and provincial-statistical data-based estimates. We found that prefectural-city-statistical data-based estimates were 60% higher than provincial-statistical data-based estimates (Fig. 7) from 2000–2010 and gradually decreased to approximately zero after 2012, i.e., the sum of prefectural-statistical data became more consistent with provincial data in recent years due to the wider coverage of large-volume industries in local statistical authorities, indicating the improvement of statistical data between these two levels.

At the prefectural city level for JJJ and other cities, the estimated differences generally increased from less than 10% to more than 300% when more inventories were included (Table 2) [5, 64–66]. Generally, the prefectural-city-statistical data-based inventories of CHRED and CEADs were more consistent than the others. For Hebei prefectural cities (such as Tangshan and Handan), the spread was relatively large among the three methods (i.e., the national, provincial and prefectural statistics data-based inventories), with differences ranging from 69 to 282 Mt CO_2_ for Tangshan and from 61 to 130 Mt CO_2_ for Handan in 2012 (Table 2). For Tangshan, the national inventories (ODIAC, EDGAR, PKU and FFDAS) were 69–117 Mt CO_2_, which were all smaller than the prefectural-city level and the provincial estimates of 138–282 Mt CO_2_ (MEIC, CHRED, MEIC and NJU). CEADs estimated Tangshan’s emissions to be much higher than the other inventories due to the large consumption of coke, e.g., the amount for 2015 was 31,986,000 tons, which was 41.4% that of Hebei Province [61]. The estimates for provincial-statistic data-based inventories were lower in Tangshan and Handan and thus tended to calculate higher emissions for other cities such as Baoding, Langfang and Hengshui due to the constraint of provincial total emissions. Moreover, the estimates for Beijing and Tianjin were more consistent for provincial estimates than the national estimates.

**Table 2:** CO_2_ emissions estimates and differences at prefectural cities in JJJ and other cities

City	Number of inventories	Year of emissions	Estimates (MtCO_2_)	(Max–min)/min (%)	References
Beijing	2	2006	115.2–160.7	39.5	Chen et al. [64]
	2	2012	112.8–113.5	0.6	Cai et al. [33]
	3	2012	79.7–134.0	68.1	Wang et al. [67]
	8	2012	82.0–254.9	210.8	This study
	8	2015	80.9–260.7	222.4	This study
Tianjin	2	2012	167.4–180.0	7.1	Cai et al. [33]
	3	2012	73.2–198.6	171.4	Wang et al. [67]
	8	2012	72.1–227.0	214.7	This study
	8	2015	80.0–231.8	189.7	This study
Shijiazhuang	4	2007	52.5–123.4	134.8	Wang et al. [67]
	8	2012	70.5–128.9	82.9	This study
	8	2015	72.0–120.3	67.1	This study
Tangshan	4	2010	47.8–188.1	293.7	Wang et al. [67]
	8	2012	69.1–281.8	307.9	This study
	8	2015	72.7–264.7	264.1	This study
Handan	5	2008	26.1–86.0	229.1	Wang et al. [67]
	2	2014	61.3–104.9	71.0	Wang et al. [67]
	8	2012	60.9–130.3	114.0	This study
	8	2015	64.5–153.1	137.4	This study
Shanghai	2	2006	179.9–189.1	5.1	Chen et al. [64]
	4	2010	112.6–420.9	273.6	Wang et al. [67]
Paris	2	2005	44.7–50.3	12.5	Chen et al. [64]
London	2	2003	28.1–32.4	15.1	Chen et al. [64]
Los Angeles	2	2000	64.5–77.6	20.4	Chen et al. [64]
Manhattan	2	2005	2.8–7.4	161.8	Chen et al. [64]
Salt Lake City	2	2011	3.2–3.8	20.8	Gurney et al. [65]
Indianapolis	2	2011	3.5–4.0	12.5	Gurney et al. [65]
Northeastern U.S. cities	4	2011	NA	50–250	Gately and Hutyra [66]

### Urban and non-urban CO_2_ emissions and implications for carbon monitoring instrument distribution

The urban extent of the ESACCI-LC product was 17%, 14%, and 5% of the land area for Beijing, Tianjin and Hebei, respectively, and the corresponding direct CO_2_ emissions were 64%, 37%, and 20%, respectively, of the total emissions for Beijing, Tianjin and Hebei for the mean of all inventories, which indicates that high emissions are spatially located more often in the urban areas of Beijing and Tianjin and are more diffusive in non-urban areas in Hebei. Similarly, an urban CO_2_ emissions study conducted by Cai et al. [33] showed that the urban extents were 17%, 17%, and 4% of land area in Beijing, Tianjin, and Hebei, which contributed 84%, 60%, and 41% of direct CO_2_ emissions, respectively. The differences between these two studies were mainly due to the inventories and urban land masks used. The ESA 300 m data showed more urban details, while Cai et al. [33] used a homemade dataset based mainly on the county/district and town/township GIS data, which are more continuous in space, to depict the urban areas. However, our study and Cai et al. [33] are largely consistent in their urban extent estimates for Beijing, Tianjin and Hebei and were approximately 20% lower than the CO_2_ emissions for urban areas calculated by Cai et al. [33]. Specifically, at the prefectural city level, emissions from national-data-based inventories were 53–75% lower in the industrial cities of Tangshan and Handan and 47–160% higher in Beijing and Tianjin than those from city-data-based inventories, and this difference was consistent with Gately and Hutyra [66]. The implication to the community and society is that national-based inventories have more biases in prefectural city levels than in local data-based inventories, and thus, we should be cautious when using national-based inventories for city-level use, such as in emissions evaluation, modeling research and policy making.

The high-emission areas recognized by most of the present inventories have significant implications for monitoring instrument deployment [7, 10, 11, 67], although this type of comparison does not allow us to further discuss the accuracy of emission estimates. Thus, this information needs to be supplemented by objective physical measurements to validate the accuracy (e.g., [14, 53]). The areas with good agreement among inventories can be key areas for observation deployment, and in return, we can use these measurements to validate the accuracy of inventories. For example, third-party monitoring of CO_2_ emissions using high-density low-cost sensor networks is becoming possible due to the development of nondispersive infrared (NDIR) technology. Martin et al. [68] investigated a low-cost NDIR sensor and compared it with the standard instrument Los Gatos and found that the accuracy could reach 2–5 ppm after environmental factor corrections. More than 300 of such CO_2_ sensors were deployed in a network in Switzerland, and they were able to resolve CO_2_ changes and differences with magnitudes larger than ~ 20 ppm [11]. Bao et al. [69] proved such sensors to be promising in high-emission areas near Shijiazhuang, Hebei Province. However, how to deploy such nodes to form an efficient network has been a challenge [9]. With emissions patterns identified by most inventories, such deployment would have more scientific references. For example, in Beijing and Tangshan, emissions from urban areas consisted of 50–70% and 20–30%, respectively, which indicated that more sensors should be deployed in the urban areas of Beijing, while more nodes should be deployed in the non-urban areas of Tangshan. Moreover, atmospheric CO_2_ measurements need to consider the combined effects of biospheric and anthropogenic signals since the JJJ region is surrounded by vegetation, especially in the western and northern parts, and atmospheric transport also plays a significant role in the measurements.

The identified high-emission areas might also indicate potential target areas for emission reductions for policy-makers if such high-emission areas are also confirmed by instruments. Beijing and Tianjin have committed to peak their CO2 emissions by 2020 and approximately 2025, respectively, in the 13th Five-Year Plan [36]. More specifically, local governments of Beijing have proposed a clear peak time of total and per capita CO_2_ emissions in 2020 in its "13th Five-Year Plan" for energy conservation and consumption reduction and climate change; thus, the city must promote the revolution in energy production and consumption, improve energy efficiency, and accelerate the construction of low-carbon cities [70]. High-emission areas (e.g., > 50,000 ton emissions in Fig. 2) need to allocate high priority to emissions control. The joint emissions control of JJJ also needs to identify the high-emission areas in Fig. 2.

### Point source contributions

**Fig. 8:**
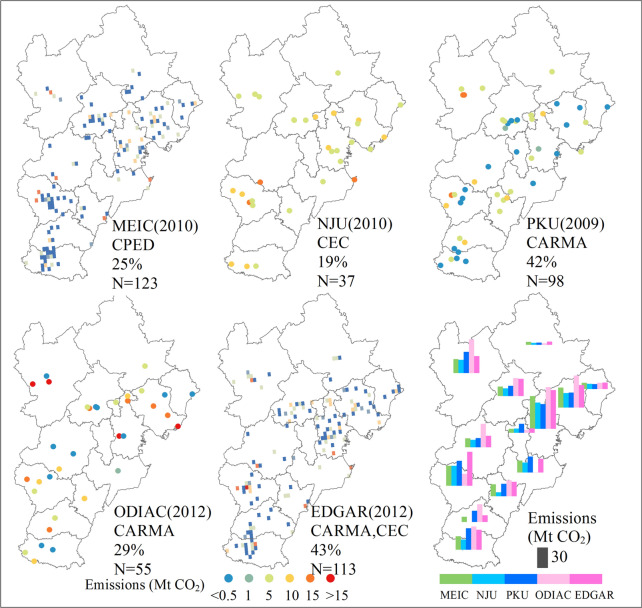
Characteristics of large emission grids and the corresponding emissions fractions and grid numbers (N)

Point emissions consist of a large proportion of total emissions [15, 33]. For the JJJ regional total, CHRED estimated the highest point emissions proportion of 78.1%, including 12,991 industrial key emission sources and 187 industrial process sites [33]. Other inventories produced much smaller proportions ranging from 19% for NJU to 43% for EDGAR (Fig. 8), and MEIC and EDGAR both had more than 100 large emission grids associated with power plants. Point sources for CHRED consisted of power plants, industries and industrial processes, while other inventories (e.g., PKU, ODIAC) mostly used the CARMA dataset, which included only power plants. NJU included power plants ranking with the top 80% in electricity production and cement production that exceeded 1 Mt yr^−1^ [47], and there were only 42 power plants and 23 cement plants and thus fewer high-emission grids (N = 37, Fig. 8), which may be the reason why NJU point emission fractions were much smaller than those of other inventories. Since the gridded maps are mostly produced based on point sources, line sources (transportation emissions) and finally area sources, and area sources are distributed using the total estimate minus the point and line sources and proxies (e.g., night light, population, GDP), point source numbers, geolocations and emission magnitudes determined a very large degree of the gridded products (e.g., [53]). To improve the accuracy in mapping emissions and mitigate the errors in emission estimates, it is important to include reliable information, preferably reported information, on large point sources as much as possible. Thus, it is preferable for the community to share the point source information and for the information to be accurate to improve the understanding of the point source emissions (e.g., [14]). This is also important for emission monitoring purposes.

## Conclusions

Here, we conducted a comprehensive analysis of city-level fossil fuel and industrial process-related CO_2_ emissions from cities in the Beijing-Tianjin-Hebei region. We showed their temporal dynamics, spatial distributions, and urban and non-urban emissions fractions. We recognized the similarities and differences in emissions and thus improved the understanding of current inventories and provided useful information for policy making in terms of reducing city emissions and monitoring CO_2_. The results showed that compared to city-data-based inventories, provincial-data-based inventories were 6% higher, and national-data-based inventories were 14% lower in 2012. Compared with city-data-based inventories, the lower estimates (53–75%) in the industrial cities of Tangshan and Handan resulted in higher estimates (47–160%) in Beijing and Tianjin for national-data-based inventories. Due to the more complete data of industrial enterprises above the state designated scale, the differences between city statistical data-based estimates and provincial statistical data-based estimates decreased from 60% to approximately zero from 2000 to 2012, indicating the improvement in local statistical authorities. Spatially, all datasets agreed with high emissions in the triangular spatial distribution pattern of Beijing-Tianjin-Tangshan and low emissions in the northern parts of Zhangjiakou and Chengde. The implications of the consistent spatial distribution patterns recognized by these inventories provide useful information for the efficient deployment of monitoring instruments, and in return, the independent measurements from these areas will increase our confidence in inventories and thus provide support for policy makers in joint emissions reductions.

## Data Availability

The data sets of ODIAC, EDGAR, PKU, CEADs and FFDAS are freely available from http://db.cger.nies.go.jp/dataset/ODIAC/DL_odiac2018.html, https://edgar.jrc.ec.europa.eu/overview.php?v=50_GHG, http://inventory.pku.edu.cn/download/download.htmlhttp://www.ceads.net/, and http://ffdas.rc.nau.edu/Data.html, respectively. CHRED, MEIC and NJU are available from the data developers upon request.

## Abbreviations

JJJ: JJJ, Beijing-Tianjin-Hebei
ODIAC: Open-source Data Inventory for Anthropogenic CO_2_
EDGAR: Emissions Database for Global Atmospheric Research
PKU: Peking University CO_2_ emission inventory
FFDAS: Fossil Fuel Data Assimilation System
CEADs: China Emission Accounts and Datasets
CHRED: China High Resolution Emission Database
MEIC: Multi-resolution Emission Inventory for China
NJU: Nanjing University CO_2_ emission inventory
SD: Standard deviation
CARMA: CARbon Monitoring for Action
CI: Confidence interval
FCPSC: The First China Pollution Source Census
CPED: China Power Emissions Database
CEC: Commission for Environmental Cooperation
ACC: China Cement Almanac
CCTEN: China Cement Industry Enterprise Indirectory
GDP: Gross domestic product
ESA: European Space Agency
CCI: Climate Change Initiative
NBS: National Bureau of Statistics
NDIR: Non-dispersive infrared
